# Nicotinamide mononucleotide adenylyltransferase promotes hypoxic survival by activating the mitochondrial unfolded protein response

**DOI:** 10.1038/cddis.2016.5

**Published:** 2016-02-25

**Authors:** X R Mao, D M Kaufman, C M Crowder

**Affiliations:** 1 Department of Anesthesiology, Washington University School of Medicine, 660 South Euclid Avenue, St Louis, MO 63110, USA; 2 Medical Scientist Training Program, Washington University School of Medicine, 660 South Euclid Avenue, St Louis, MO 63110, USA; 3 Department of Anesthesiology and Pain Medicine, University of Washington School of Medicine, 1959 NE Pacific Street, Seattle, WA 98195-6540, USA; 4 Department of Genome Sciences, University of Washington School of Medicine, 1959 NE Pacific Street, Seattle, WA 98195-6540, USA

## Abstract

Gain-of-function mutations in the mouse nicotinamide mononucleotide adenylyltransferase type 1 (Nmnat1) produce two remarkable phenotypes: protection against traumatic axonal degeneration and reduced hypoxic brain injury. Despite intensive efforts, the mechanism of Nmnat1 cytoprotection remains elusive. To develop a new model to define this mechanism, we heterologously expressed a mouse Nmnat1 non-nuclear-localized gain-of-function mutant gene (m-nonN-Nmnat1) in the nematode *Caenorhabditis elegans* and show that it provides protection from both hypoxia-induced animal death and taxol-induced axonal pathology. Additionally, we find that m-nonN-Nmnat1 significantly lengthens *C. elegans* lifespan. Using the hypoxia-protective phenotype in *C. elegans*, we performed a candidate screen for genetic suppressors of m-nonN-Nmnat1 cytoprotection. Loss of function in two genes, *haf-1* and *dve-1*, encoding mitochondrial unfolded protein response (mitoUPR) factors were identified as suppressors. M-nonN-Nmnat1 induced a transcriptional reporter of the mitoUPR gene *hsp-6* and provided protection from the mitochondrial proteostasis toxin ethidium bromide. M-nonN-Nmnat1 was also protective against axonal degeneration in *C. elegans* induced by the chemotherapy drug taxol. Taxol markedly reduced basal expression of a mitoUPR reporter; the expression was restored by m-nonN-Nmnat1. Taken together, these data implicate the mitoUPR as a mechanism whereby Nmnat1 protects from hypoxic and axonal injury.

About 25 years ago, Lunn *et al.*^1^ reported a remarkable mouse strain (later named *wlds*) where Wallerian axonal degeneration after traumatic nerve injury was markedly reduced. The responsible genetic lesion in the *wlds* strain was found to be a translocation and triplication that produced a mutant fusion protein consisting of a segment of an E4-ubiquitin ligase-like protein and full-length Nmnat1, a primarily nuclear-localized nicotinamide adenine dinucleotide (NAD) biosynthetic enzyme.^2, 3, 4^ Subsequently, overexpression of Nmnat1 or a non-nuclear-localized mutant form of Nmnat1 (nonN-Nmnat1) was shown to be sufficient to prevent axonal degeneration in mice.^5^ The mechanism whereby Nmnat1 is axonal protective has remained elusive, although substantial evidence points to a mitochondrial site of action.^6, 7, 8^ Recently, a transgenic mouse with the expression of nonN-Nmnat1 was found to have reduced brain injury in a model of neonatal hypoxic/ischemic stroke.^9^ Although the reduced stroke volume might be secondary to a reduced axonal degeneration, this result suggests the hypothesis that nonN-Nmnat1 may have broader cytoprotective properties.

*Caenorhabditis elegans* has become an increasingly important model for the study of both hypoxic and axonal injury.^10, 11, 12, 13, 14, 15, 16^ In this regard, the strengths of the model lie in its genetic tractability, fully defined anatomy and cellular identity, and the ability to directly observe cell pathology in live animals. Screens in *C. elegans* for genes that control hypoxic sensitivity have implicated multiple distinct pathways as determinants of hypoxic death.^16, 17, 18, 19^ In particular, genetic perturbations that improve cellular proteostasis are generally hypoxia protective.^16, 17, 18, 19, 20, 21^ These findings argue that hypoxia perturbs protein folding and that this perturbation contributes to cell death; indeed, substantial evidence in *C. elegans* and in other models indicates that hypoxia/ischemia disrupts protein folding homeostasis.^16, 17, 19, 22, 23^

In this work, we set out to answer two outstanding questions about Nmnat1. First, is hypoxia protection a general feature of Nmnat1 expression or is it peculiar to the mouse transgenic model previously tested? Second, what is the mechanism whereby Nmnat1 protects from hypoxia? For the first question, we expressed the mouse non-nuclear-localized-Nmnat1 (m-nonN-Nmnat1) in two distinct settings, primary mouse hippocampal neurons and in *C. elegans*, and asked whether in these contexts m-nonN-Nmnat1 was hypoxia protective. Additionally, we overexpressed the *C. elegans* homologs of Nmnat1 to determine if other isoforms of Nmnat1 were hypoxia protective. For the second question, we used the *C. elegans* model to test a variety of candidate pathways for their role in Nmnat1 hypoxia protection.

## Results

### Mouse nonN-Nmnat1 protects mouse primary hippocampal neuron cultures from hypoxic injury

**Figure 1:**
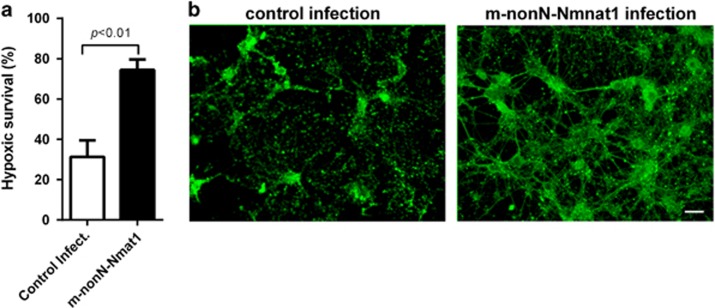
m-nonN-Nmnat1 protects primary mouse hippocampal neurons from hypoxic injury. (**a**) Hypoxic survival of primary mouse hippocampal neurons infected with lentivirus carrying m-nonN-Nmnat1 or control luciferase. Values are mean±S.E.M.; *P*<0.01 by unpaired *T*-test of four trials. (**b**) Representative fluorescent photomicrographs of hypoxia-exposed hippocampal neurons with and without m-nonN-Nmat1 infection. Neurons were visualized with Tuj1 antibody. Scale bar, 10 *μ*m

We first determined whether mouse nonN-Nmnat1 directly protects neurons from hypoxic injury as our primary interest is neuronal hypoxic injury and as the previous study showed Nmnat1 hypoxic protection in a transgenic mouse model where anti-inflammatory effects or genetic background contributions cannot be ruled out.^9^ Using a lentiviral expression system,^24^ we expressed m-nonN-Nmnat1 in primary mouse hippocampal neuron cultures essentially devoid of other cell types and found that m-nonN-Nmnat1 indeed provides strong hypoxic protection to neurons (Figure 1).

### Mouse nonN-Nmnat1 protects *C. elegans* from hypoxic injury

**Figure 2:**
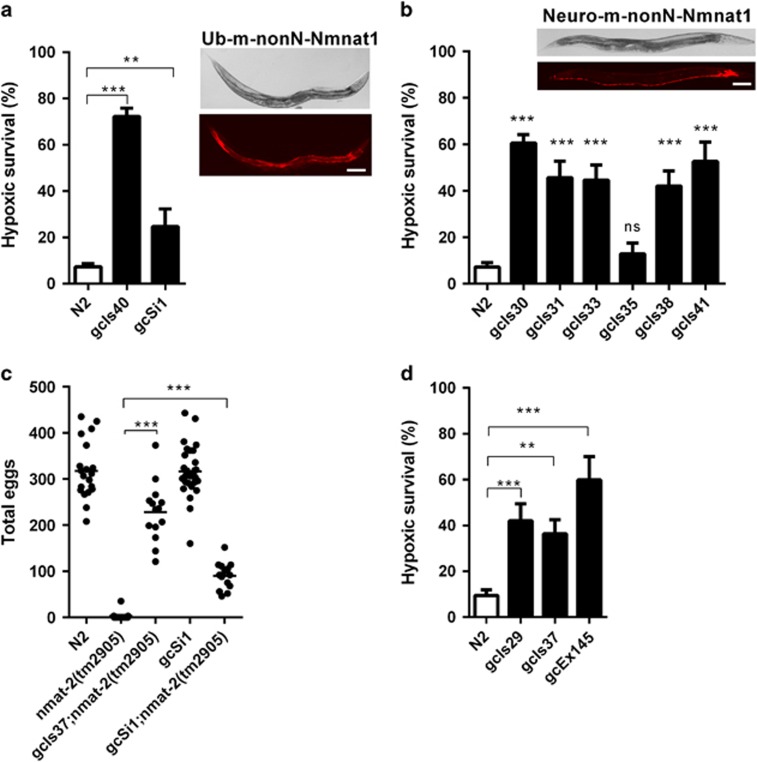
m-nonN-Nmnat1 protects worms from hypoxia. (**a**) Hypoxic survival of ubiquitous-expressing ub-m-nonN-Nmnat1 strains. High copy – *gcIs40* and single copy – *gcSi1* integrants of the P*rpl-28*::mCherry::m-nonN-Nmnat1 transgene were tested. (***P<0.0001, **P<0.01, NS, not significant – unpaired *T*-test, mean±S.E.M. shown, *n*=15 trials for N2(wild type) and *gcIs40*, *n*=6 for *gcSi1*). Inset: *gcIs40* worms (top – DIC; bottom – mCherry filter). (**b**) Hypoxic survival of pan-neuronal-expressing neuro-m-nonN-Nmnat1 strains (P*rab3*::mCherry::m-nonN-Nmnat1) (trials *n*=5–20). Inset: *gcIs30* worms (top – DIC; bottom – mCherry filter; scale bars for (**a**) and (**b**)=100 *μ*m). (**c**) The infertility of *nmat-2(tm2905)* could be rescued by ubiquitous *C. elegans nmat-1* overexpression (*gcIs37*) and m-nonN-Nmnat1 expression (*gcSi1*) (each data point represents the total brood from one animal). (**d**) Hypoxic survival of *C. elegans nmat-1* and *nmat-2* overexpression. *gcEx145* contains an extrachromosomal *nmat-1* array [P*rpl-28::nmat-1*::mCherry] *gcIs29* and *gcIs37* contain integrated *nmat-2* arrays (P*rpl-28::nmat-2*::mCherry) (trials: *n*=12, 7, 12 and 3 for N2, *gcIs29*, *gcIs37* and *gcEx154*, respectively)

Having demonstrated definitively that m-nonN-Nmnat1 protected mouse neurons from hypoxia, we wanted to know if this phenotype extended across phyla and therefore is likely a general property of Nmnat1. Towards this end, we expressed m-nonN-Nmnat1 heterologously in the nematode *C. elegans* (Table 1). As in mouse, m-nonN-Nmnat1 expression in *C. elegans* was strongly protective against hypoxic injury. Strains with ubiquitous expression in all *C. elegans* cells (ub-m-nonN-Nmnat1) were hypoxia resistant with even a single copy insertion conferring significant hypoxic protection (Figure 2a). Given the primarily neuronal phenotype in mouse, we asked whether m-nonN-Nmnat1 expression in neurons alone (neuro-m-nonN-Nmnat1) is sufficient to protect the organism from hypoxia and found that neuronal expression did indeed provide strong protection (Figure 2b). We examined the relative level of expression of the various transgenes, in particular to determine why one of six neuro-m-nonN-Nmnat1 strains had only a low, nonstatistically significant level of protection. The level of protection correlated for neuronal transgenes with the m-nonN-Nmnat1 transcript levels consistent with its gain-of-function activity (Supplementary Figure S1). We next asked whether the m-nonN-Nmnat1 transgene was functioning like the native *C. elegans* Nmnat1 and found that ub-m-nonN-Nmnat1 could rescue the sterility phenotype of a loss-of-function mutant of *nmat-2*, one of the two *C. elegans* Nmnat1 homologs (Figure 2c).^25^ Similarly, ubiquitous overexpression of *nmat-2* and the other *C. elegans* Nmnat1 homolog *nmat-1* both provided significant hypoxic protection (Figure 2d). Thus, hypoxia protection is a conserved phenotype of both worm and mouse Nmnat1 gain-of-function.

### M-nonN-Nmnat1 does not affect global oxygen consumption in *C.elegans*

Given that Nmnat1 is a biosynthetic enzyme for NAD, a general mechanism of Nmnat1 hypoxia protection that we considered was that the m-nonN-Nmnat1 expression reduces oxygen consumption and thereby reduces the duration of cellular hypoxia and severity of injury. However, oxygen consumption was similar to wild type in both m-nonN-Nmnat1 and *nmat-2* overexpression animals (Supplementary Figure S2). Thus, the observed Nmnat1 hypoxia protection is not because of a simple oxygen conservation mechanism.

### Mitochondrial unfolded protein response genes are required for m-nonN-Nmnat1 hypoxia resistance

**Figure 3:**
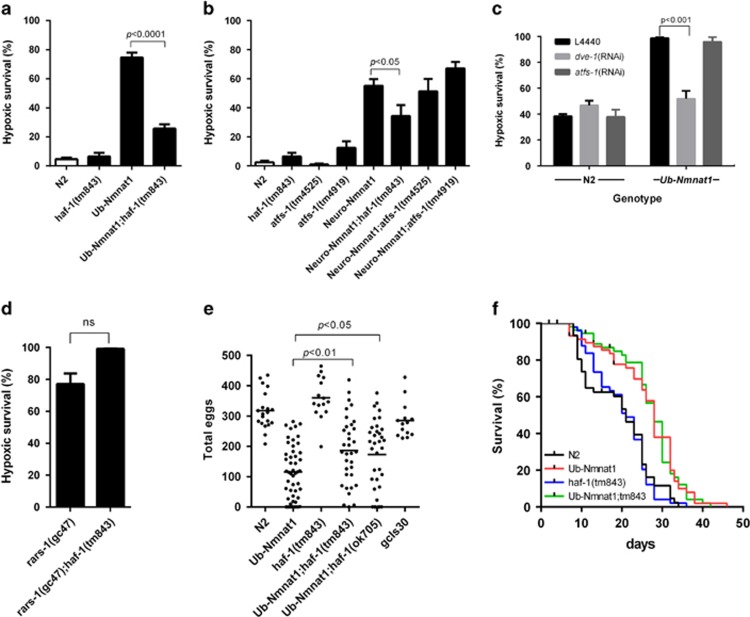
Mitochondrial UPR components are required for m-nonN-Nmnat1 phenotypes. (**a** and **b**) Loss-of-function mutations of *haf-1* but not *atfs-1* suppressed the hypoxia resistance of Ub-Nmnat1(*gcIs40*[ub-m-nonN-Nmnat1]) (**a**) and Neuro-Nmnat1(*gcIs30*[neuro-m-nonN-Nmnat1] (**b**) (mean±S.E.M., unpaired *T*-test, *n*=5–10 trials). (**c**) RNAi against *dve-1* but not *atfs-1* suppressed Ub-Nmnat1(*gcIs40*[Ub-m-nonN-Nmnat1]) hypoxia resistance. Hypoxic treatment was for 18 h (mean±S.E.M., unpaired *T*-test, *n*=3 trials). (**d**) Loss of function of *haf-1* did not suppress *rars-1(gc47)* hypoxia resistance; hypoxic treatment was for 30 h (mean±S.E.M., unpaired *T*-test, *n*=3 trials). (**e**) Mutation of *haf-1* rescued the low fertility of Ub-Nmnat1[*gcIs40*(Ub-m-nonN-Nmnat1]) (*n*=14–49, unpaired *T*-test, N2 *versus* Ub-Nmnat1 – *P*<0.0001; N2 *versus* Neuro-Nmnat1(*gcIs30*[Neuro-m-nonN-Nmnat1] – *P*=0.13. (**f**) Worm lifespan was significantly extended by Ub-Nmnat1(*gcIs40*[ub-m-nonN-Nmnat1] (*P*<0.0001, N2 *versus gcIs40*, log-rank test); the lifespan extension was not significantly suppressed by *haf-1(tm843)*

We tested a variety of mutant genes that might plausibly have a phenotypic interaction with m-nonN-Nmnat1. Most genes that were tested had no effect on the hypoxia-protective phenotype (Supplementary Figure S3). However, we found that loss-of-function mutations in the gene *haf-1*, which encodes a mitochondrial outer membrane ABC transporter previously shown to function as an activator of the mitochondrial unfolded protein response (mitoUPR),^26^ significantly suppressed the hypoxia resistance of both ub-m-nonN-Nmnat1 and neuro-m-nonN-Nmnat1 transgenes (Figures 3a and b). The genes *dve-1* and *atfs-1* also function in the *C. elegans* mitoUPR, encoding transcription factors that promote the expression of the mitochondrial-specific chaperones HSP-6 and HSP-60.^26,27,28,29^ Similar to *haf-1*(lf) mutants, *dve-1* double-stranded RNA-mediated interference (RNAi) suppressed the m-nonN-Nmnat1 hypoxia-resistant phenotype (Figure 3c). Interestingly, neither loss-of-function mutations nor RNAi knockdown of *atfs-1*, a gene believed to be required for mitoUPR induction under most conditions, suppressed m-nonN-Nmnat1-mediated hypoxia protection (Figures 3b and c), demonstrating that m-nonN-Nmnat1 is activating the mitoUPR in a non-*atfs-1*-dependent manner. Several mitochondrial-resident proteases are thought to function upstream of the peptide transporter HAF-1.^27, 29^ Mutations in these genes greatly compromised worm development and rendered data from the double mutations difficult to interpret. Nevertheless, in cases where the double mutant animals were healthy enough for hypoxia testing, none of these mutations suppressed m-nonN-Nmnat1 hypoxia protection (Supplementary Figure S4a). Thus, the suppression of the hypoxia-resistant phenotype of m-nonN-Nmnat1 is not a general property of mitoUPR mutants but is relatively specific to *haf-1* and *dve-1.* Mutations in ABC transporters for other organelles such as *haf-2* and *haf-3* did not suppress m-nonN-Nmnat1 protection against hypoxia (Supplementary Figure S4b). Additionally, the suppression by the *haf-1* is specific to Nmnat1 hypoxia resistance in that *haf-1*(lf) did not suppress the hypoxic protection produced by a previously identified hypoxia-resistant allele of the arginyl tRNA synthetase gene – *rars-1*(*gc47*) (Figure 3d).^17^

### M-nonN-Nmnat1 worms are long lived

While generally characterizing the m-nonN-Nmnat1 strains, we found that the strongly expressing ub-m-nonN-Nmnat1 strain *gcIs40* had two additional phenotypes, reduced fertility and long lifespan (Supplementary Table S1). We asked whether these Nmnat1 phenotypes were regulated by the same pathways as hypoxic injury. Both *haf-1* mutants strongly suppressed the infertility phenotype of ub-m-nonN-Nmnat1 (Figure 3e and Supplementary Table S2). However, surprisingly, *haf-1(tm843)* did not suppress lifespan extension (Figure 3f). It is important to point out that infertility was not causally related to the hypoxia resistance phenotype of m-nonN-Nmnat1 in that *gcSi1*, a single copy insertion allele of ub-m-nonN-Nmnat1, is hypoxia resistant but has normal fertility (Figures 2a and c), and several of the hypoxia-resistant neuro-m-nonN-Nmnat1 strains have normal fertility (Figures 2b and Supplementary Table S1). The lack of concordance of the suppression of the lifespan extension and infertility phenotypes in the *haf-1(lf);gcIs40* mutants argues that the underlying mechanisms of hypoxia resistance and lifespan extension by Nmnat1 are also distinct.

### M-nonN-Nmnat1 protects from mitochondrial proteostasis stress

**Figure 4:**
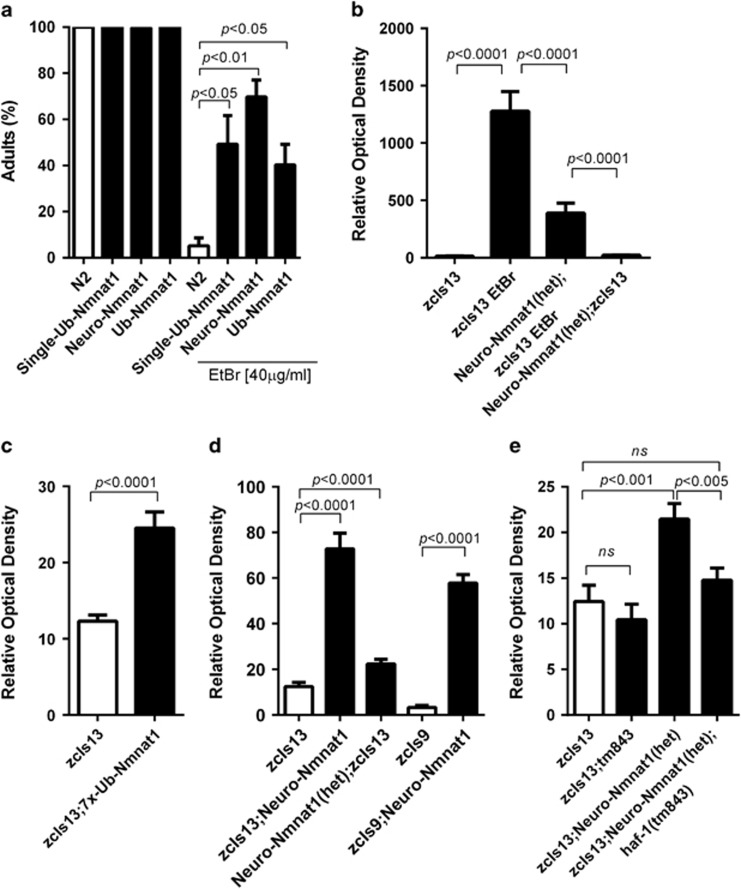
m-nonN-Nmnat1 protects from mitochondrial folding stress and activates the mitoUPR. (**a**) m-nonN-Nmnat1 protected worms from EtBR-induced developmental arrest. Eggs were laid on plates with or without EtBr (40 *μ*g/ml) and percent adults was scored 4 days later (mean±S.E.M., unpaired *T*-test; *n*=4 trials). (**b**) The induction of the mitoUPR reporter P*hsp-6*::GFP (*zcIs13*) by EtBr was significantly reduced by Neuro-Nmnat1(*gcIs41*[Neuro-m-nonN-Nmnat1]) (mean±S.E.M., unpaired *T*-test; *n*=28–50 animals per condition); note gcIs41 heterozygotes were used because of difficulty constructing *zcIs13;gcIs41* homozygotes. (**c** and **d**) Under basal conditions, both Ub-Nmnat1(*gcSi6;gcSi7*[7X-Ub-m-nonN-Nmnat1] and Neuro-Nmnat1(*gcIs41*[Neuro-m-nonN-Nmat1]) activate mitoUPR reporter *zcIs13*[P*hsp-6*::GFP] Neuro-Nmnat1(*gcIs41*[Neuro-m-nonN-Nmat1]) also activates *zcIs9*[P*hsp-60*::GFP]. (**e**) *haf-1*(*tm843*) suppressed Neuro-Nmnat1(*gcIs41*[neuro-m-nonN-Nmnat1])-induced mitoUPR (for (**b–e**) mean±S.E.M., unpaired *T*-test; three trials with at least 15 worms imaged for each genotype/condition)

The suppression phenotypes by *haf-1* and *dve-1* loss-of-function suggests that m-nonN-Nmnat1 may ameliorate mitochondrial proteostasis stress. To test this hypothesis, we measured the sensitivity of m-nonN-Nmnat1 to ethidium bromide (EtBr), which produces mitochondrial protein folding stress.^30^ Both neuronal and ubiquitous m-nonN-Nmnat1 strains were highly protective against the toxic effects of EtBr (Figure 4a). Consistent with amelioration of mitochondrial folding stress rather than other effects of EtBr on mitochondria such as suppressing transcription, neuro-m-nonN-Nmnat1 significantly reduced the induction by EtBr of the mitoUPR transcriptional reporter, P*hsp-6*::GFP(*zcIs13*) (Figure 4b).^30^ Surprisingly, in the absence of EtBr, both neuro- and ub-m-nonN-Nmnat1 mildly but significantly increased the basal level of activation of P*hsp-6*::GFP, and neuro-m-nonN-Nmnat1 increased P*hsp-60*::GFP (Figures 4c and d). The results suggest that the protective effect of m-nonN-Nmnat1 may be hormetic in nature, by producing a low level of mitochondrial proteostasis stress that induces a mitoUPR-dependent protective response. Consistent with this hypothesis, basal mitoUPR induction by m-nonN-Nmnat1 was significantly suppressed by *haf-1*(lf) (Figure 4e). Moreover, expression of m-nonN-Nmnat1 in mouse primary hippocampal neurons could induce the expression of mitochondrial chaperone hspa9 (closest hsp-6 homolog in mouse); however, no induction of hspd1 was observed (closest hsp-60 homolog) (Supplementary Figure S5). An enzymatic activity compromised version of m-nonN-Nmnat1 (H24A)^24^ had not effect on the expression of hspd1, demonstrating that enzymatic activity of nonN-Nmnat1 is required for induction in mouse neurons.

### Taxol-induced axonal degeneration ameliorated by m-nonN-Nmnat1

**Figure 5:**
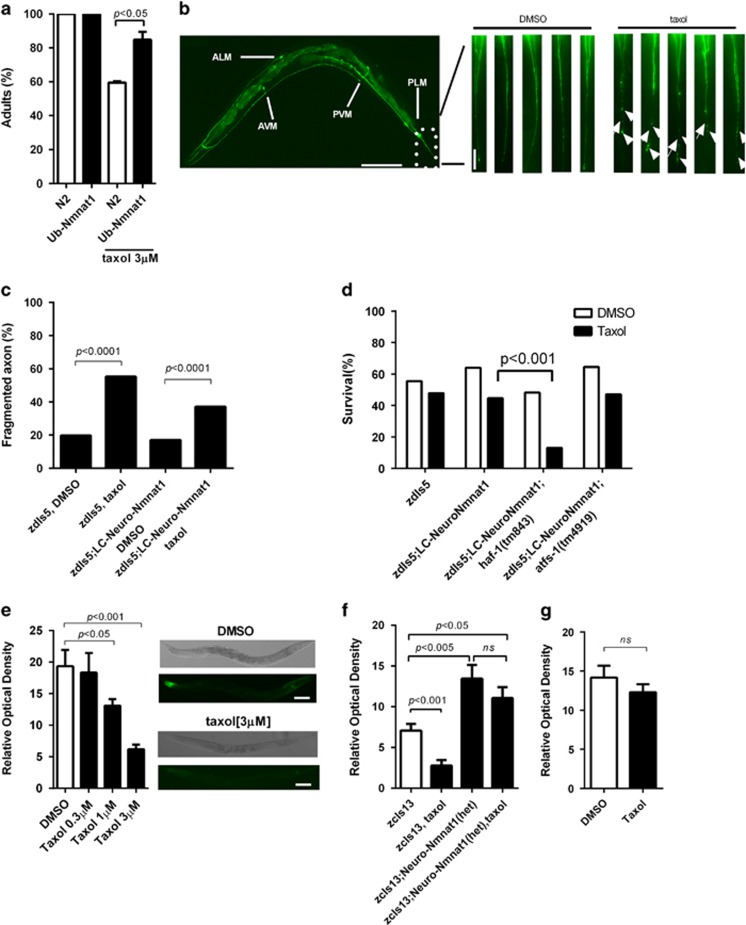
m-nonN-Nmnat1 reduces axonal degeneration induced by taxol. (**a**) Taxol-mediated *C. elegans* developmental arrest is rescued by Ub-Nmnat1(*gcIs40*[ub-m-nonN-Nmnat1]) (mean±S.E.M., unpaired *T*-test, three trials). Eggs were laid on taxol plates and four days later the number of adults was scored. (**b**) Worm mechanosensory neurons and axons labeled by *zdIs5*[*mec-4*::GFP] (scale bar: 100 *μ*m; scale bar in inset: 10 *μ*m). Representative images from the tail region comparing DMSO buffer *versus* taxol. Note fragmented (arrow) and beaded (arrowhead) axons in the taxol condition. (**c**) Taxol-induced axon degeneration was rescued by Neuro-Nmnat1[*gcIs35*(neuro-m-nonN-Nmnat1]) (Fisher's exact test, 141–494 axons were scored in five independent trials and pooled together). (**d**) *haf-1(tm843*) reduced survival of NeuroNmnat1 animals (*gcIs35*[neuro-m-nonN-Nmnat1]) in the presence of chronic taxol but *atfs-1(tm4919)* did not. Eggs were laid on taxol (3 *μ*M) plates and at 15 days after adulthood, surviving worms were scored (Fisher's exact test; data were pooled from four independent trials, *n*=239–314). (**e**) Taxol acutely reduced basal mitoUPR reporter *zcIs13*[P*hsp-6*::GFP] activity). Inset: worms under DIC (top) and GFP filter (bottom, scale bar: 100 *μ*m). (**f**) Heterozygous Neuro-Nmnat1(*gcIs41*[Neuro-m-nonN-Nmnat1]) preserved mitoUPR reporter activity following taxol (3 *μ*m) treatment (mean±S.E.M., unpaired *T*-test; three independent trials). (**g**) Taxol did not alter the expression of the ER-UPR reporter activity *zcIs4* [P*hsp-4*::GFP] (mean±S.E.M., unpaired *T*-test; three independent trials)

Finally, we asked whether m-nonN-Nmnat1 protected against axonal degeneration in *C. elegans*, the original phenotype of the *wlds* mouse. Worm axon degeneration has been reported in spectrin *unc-70* mutants and in a model of necrosis-induced neuron degeneration,^25, 31^ and overexpression of worm *nmat-2* was shown to be protective against degeneration of mechanosensory neurons.^25^ To examine the role m-nonN-Nmnat1 in axon degeneration, we used taxol, a chemotherapeutic agent, previously shown to induce axonal degeneration in cancer patients and in experimental models.^32, 33, 34^ Worms hatched from taxol-containing plates had significant growth retardation that could be rescued by ub-m-nonN-Nmnat1 (Figure 5a). To observe axonal degeneration directly, GFP-labeled mechanosensory neurons were examined after taxol treatment. In elderly animals, mechanosensory axonal pathology manifesting as axonal beading and gaps, particularly in the PLM neurons in the tail of the animals, was seen (Figure 5b) and was significantly increased in taxol-treated animals (Figure 5c). The taxol-induced axonal degeneration was significantly reduced by the neuro-m-nonN-Nmnat1 transgene *gcIs35* (Figure 5c); the low expressing *gcIs35* transgene was used because it did not extend lifespan, thereby avoiding a potential confounding factor (Supplementary Figure S6 and Supplementary Table S2). Despite initial developmental growth retardation, taxol did not alter adult lifespan in wild-type animals (Supplementary Figure S6 and Supplementary Table S2). However, taxol was lethally toxic to elderly *haf-1* but not *atfs-1* loss-of-function animals (Figure 5d). These data suggest the hypotheses that taxol produces mitochondrial folding stress or that it affects the *haf-1-*dependent induction of the mitoUPR in response to mitochondrial proteostatic stress. Consistent with the latter hypothesis, taxol dose dependently reduced the basal expression of the P*hsp-6::GFP* mitoUPR reporter and this reduction of the expression of the mitoUPR reporter was blocked by neuronal m-nonN-Nmnat1 (Figures 5e and f). The analogous ER unfolded protein response reporter P*hsp-4::*GFP^35^ was unaffected by taxol, indicating relative specificity for the mitoUPR (Figure 5g).

## Discussion

Nmnat1 has been found to protect from axonal degeneration secondary to a variety of insults.^36, 37, 38, 39^ Most recently, a transgenic mouse strain expressing the same non-nuclear-localized Nmnat1 protein used in this study was found to have a reduced stroke volume following a hypoxic ischemic insult.^9^ Unlike previous work, the Verghese *et al.*^9^ study suggests that nonN-Nmnat1 protects neuronal cell bodies from acute injury, not just axons from delayed degeneration. Our results in both mouse primary neurons and in *C. elegans* definitively confirm the hypoxia-protective activity of non-nuclear-localized Nmnat1. Our data also show that hypoxia protection is not peculiar to m-nonN-Nmnat1 but rather is a general feature of Nmnats since overexpressions of two *C. elegans* Nmnat homologs also improve hypoxic survival. Additionally, our work uncovered a novel nicotinamide mononucleotide adenylyltransferase (Nmnat) stress resistant phenotype – prolonged lifespan. Our data show that m-nonN-Nmnat1 activates the mitoUPR. Given that the mitoUPR has been implicated in lifespan extension in *C. elegans* and other animals,^40^ it is not particularly surprising that m-nonN-Nmnat1 extends lifespan in *C. elegans.* Further, the lifespan extension phenotype shows that nonN-Nmnat1 is not only protective against the acute high level stress of hypoxia and traumatic nerve injury but also against the indolent stress of aging.

Despite considerable effort, the mechanism whereby nonN-Nmnat1 protects from axonal degeneration, much less the mechanism of any of its more recently defined phenotypes, has not been defined. However, mitochondria have been repeatedly implicated as having a role. The mitochondrially localized mammalian isoform Nmnat3 has been shown to phenocopy the axonal degeneration-protective activity of the Nmnat1 *wlds* mutant, whereas simple overexpression of the nuclear-localized Nmnat1 or the golgi-localized Nmnat2 does not protect from axonal degeneration.^8, 41^ The axonal-protective phenotype correlates with nonN-Nmnat1 expression in the mitochondria matrix and mitochondrial ATP content has been found to be higher in Nmnat3 and *wlds* mice compared to wild-type mice.^8^ Nmnat3 heterologous expression in *Drosophila* was also protective against axotomy-induced axonal degeneration, and as in mammalian cells, Nmnat3 localizes to fly mitochondria.^6, 42^ Knockdown of endogenous *Drosophila* nicotinamide mononucleotide adenylyltransferase (dNmnat) in fly results in a rapid loss of axonal mitochondria, suggesting that dNmnat is required for mitochondrial health.^43^ Expression of the *wld*s protein in fly results in an increase in mitochondrial calcium buffering capacity resulting in improved axonal mitochondrial motility.^42^ Whether improved mitochondrial calcium buffering capacity, improved mitochondrial proteostasis or a combination of the two is responsible for Nmnat-mediated stress protection is unclear. However, we recently reported that hypoxia disrupts mitochondrial proteostasis and that induction of the mitoUPR by genetic or pharmacological agents was hypoxia protective in *C. elegans*.^44^ Thus, we favor improved mitochondrial proteostasis by Nmnat as a primary mediator of hypoxic survival.

The enzymatic product of Nmnat1, NAD, has been shown to be axonal-protective^5^ and to activate the mitoUPR to maintain mitochondrial proteostasis.^45^ Further NAD and its precursors have been found to extend lifespan in *C. elegans* and multiple other models.^46^ Even though overall NAD levels have been found to be unchanged in nonN-Nmnat1 transgenic mice,^24^ the enzymatic activity of Nmnat1 is required for its axonal-protective phenotype in mouse and for its hypoxia-protective phenotype in mouse primary neuron cultures (Supplementary Figure S5). Mammalian mitochondrial NAD has to be synthesized *in situ* by the mitochondrial-specific isoform Nmnat3;^47^ perhaps, nonN-Nmnat1 similarly increases mitochondrial NAD levels without affecting overall cellular NAD. Therefore, our working hypothesis based on these data is that m-nonN-Nmnat1 maintains mitochondrial NAD levels and thereby supports mitoUPR function and mitochondrial proteostasis in injured neurons and presumably other cell types to prevent degeneration and cell death.

## Materials and Methods

### Strains

*C. elegans* strains were obtained from the *Caenorhabditis* Genetics Center and Japan National BioResource Project and outcrossed three times before testing. Mutations were confirmed after outcrossing by PCR. All strains were maintained at 20 °C on nematode growth media agar seeded with OP50 bacteria as described previously.^48^ Information for all alleles used is available at www.wormbase.org, and all mutations were consistent with the published information at Wormbase. New transgenic strains are listed in Table 1. Double and triple mutant strains were generated by standard genetic methods,^49^ and genotypes were confirmed by phenotype and/or PCR. RNAi experiments were performed as described previously.^18^

### Transgenic animals

*nmat-1* and *nmat-2* cDNA was amplified from wild-type N2 and confirmed by sequencing. The *rpl-28* promoter (1.4 kb) was subcloned from the Andy Fire Vector Kit (pPD129.57; Addgene, Cambridge, MA, USA). An expression construct with the *rab-3* promoter was a gift from Mike Nonet (Washington University School of Medicine, St Louis, MO, USA).^50^ Mouse nonN-Nmnat1 cDNA was a gift from Jeffery Milbrandt (Washington University School of Medicine) and was tagged with mCherry and 6X-His.^24^ P*rpl-28*::mCherry::m-nonN-Nmnat1, P*rpl-28*::*nmat-1*::mCherry, P*rpl-28*::*nmat-2*::mCherry and P*rab3*::m-nonN-Nmnat1::mCherry were injected into N2 gonads along with the coinjection marker pPHGFP (except gcIs41, which was without coinjection marker).^51^ Transgenic animals were selected by the expression of fluorescent markers. To make integrated lines, 30–40 young adult worms carrying transgenes were UV irradiated as described previously.^51^ At least 100 F1s with expression from the transgene were cloned and lines where 100% of animals stably expressed the transgene were kept and outcrossed at least 10 times.

Single copy chromosomal insertions were produced as described previously.^52^ Single copy (gcSi1), two tandem copy (gcSi3) and four tandem copy (gcSi6) of P*rpl-28*::m-nonN-Nmnat1 was inserted into ttTi5605 II site by the MosSCI method, whereas three tandem copies (gcSi7) was inserted into the cxTi10882 IV site.^52^ Insertion sites and integrity of the integrated sequences were verified by PCR and restriction mapping.

### *C. elegans* hypoxia assays

Hypoxic incubation and scoring of *C. elegans* was performed as described previously.^18^ For each trial, three plates of young adult worms (one day after L4 worms, 30–60 worms per plate) were used. After hypoxic treatment, worms from these three plates were scored after a 24-h recovery as alive or dead, and the values from the three plates were pooled as a single trial. Hypoxic incubation was for 20 h, unless otherwise stated. Numbers of trials are listed in the individual figure legends.

### Fertility tests

L4 worms (14–20 for each genotype) were placed in plates individually. The worms were transferred to new plates and eggs were counted daily until no eggs were laid for two consecutive days. Worms burrowing, bagging or desiccated were censored from analysis.

### Lifespan analysis

Lifespan assays were conducted at 20 °C as described previously.^53^ After the L4 molting, animals were transferred to plates containing 100 *μ*M 5-fluoro-2′-deoxyuridine (FUDR; Sigma). We used the L4 molt as *t*=0 for lifespan analysis. Strains were grown at 20 °C for at least five generations before lifespan determination. Exploded, bagged and desiccated worms were censored.

### EtBr and taxol developmental assay

EtBr (40 *μ*g/ml) or taxol (3 *μ*M) were added to plates 24 h before synchronization. Eggs (30–50/plate, three plates/genotype/trial) were allowed to hatch on EtBr or taxol plates. The adult animals were counted 4 days after hatching and divided by total eggs to obtain percent of adults.

### O_2_ consumption

Oxygen consumption assays were performed as reported previously.^16^ At least 3000 young adults were used for each test for each genotype and the experiments were repeated at least three times.

### GFP reporter assays

Worms were mounted on 2% agarose pads and immobilized using 10 *μ*M levamisole in M9. All images were taken using a Zeiss Axioskop 2 microscope (Carl Zeiss Microscopy, Jena, Germany) with the x10 objective and a Retiga EXi Fast1394 digital camera (QImaging, Surrey, BC, Canada). At least 15 animals were used for each trial, with at least three trials for each genotype/treatment. Fluorescent intensities were measured and analyzed with ImageJ software (NIH, Bethesda, MD, USA) as described previously.^17^

### Taxol and axon degeneration

Paclitaxel/taxol was purchased from Sigma (T7402) and dissolved in dimethyl sulfoxide (DMSO; Sigma D2650) to make a 10 mM stock solution and stored at −80 °C. The stock solution was diluted immediately before use into DMSO to make 0.6 mM intermediate solution, 50 *μ*l of which was added to NGM plates (10 ml) the night before use to make final 3 *μ*M taxol assay plates. Control plates contained 50 *μ*l DMSO. The 0.3 and 1 *μ*m plates were made similarly. Worm populations were synchronized on these plates and 100 *μ*M mitotic inhibitor FUDR (F0503; Sigma) was added into plates once worms grew into young adults. When the food became scarce, worms were picked onto new plates with the same conditions. All surviving day 15 adults (L4=day 0) were picked to examine axon phenotypes under a compound microscope (Zeiss Axioskop2 plus, Zeiss North America, Thornwood, NY, USA). Pictures of all PLM posterior processes were taken by using a x63 objective and Photoshop software (Adobe Systems, San Jose, CA, USA) was used for further image processing such as rotating and adding pseudocolor. An axon was scored as pathological when clear gaps, truncation and beading were present. The images were scored blinded to condition. Only the posterior processes of PLM neurons were found to have reproducible axon degeneration. Sporadic degenerations at the anterior of PLM and in the ALM neurons was rarely observed.

### RT-qPCR

The detailed protocol has been described previously.^19^ Total RNA was extracted from worms or primary neurons with TRIzol LS reagent (Life Technologies, Carlsbad, CA, USA; no. 10296-010). Primers for m-nonN-Nmnat1 (all nonN-Nmnat1 constructs were mCherry and 6 × his tagged): forward, 5′-ACTGGAAAAGCCTGGGGCGGCGGCGGC-3′ and reverse, 5′-ATGGTGATGGTGATGCAAAGTGGAATGG-3′; *β*-actin: forward, 5′-GACATGGAGAAGATCTGGCA-3′ and reverse, 5′-GGTCTCAAACATGATCTGGGT-3′; hspa9: forward, 5′-CAGGAAGAAGGAACGTGTTG-3′ and reverse, 5′-GCTTGTTGCACTCATCAGCAG-3′ hspd1: forward, 5′-CCTGTGACAACCCCTGAAG-3′ and reverse, 5′-CACTCAACAAGACATAGGCA-3′. qPCR (SYBR green) was run at 95 °C for 15 s and 60 °C for 30 s and analyzed using the ΔΔC_T_ method.

### Hippocampal neurons and hypoxic treatment

Primary P0 mouse hippocampal neurons were established in poly-d-lysine-coated 4-well dishes with Neurobasal medium supplemented with B27 (Life Technologies; 17504-044), l-glutamine and FUDR to prevent glial growth. After 5 days *in vitro*, the neurons were infected with lentivirus containing sequence encoding the desired proteins.^24^ Five days after infection, nonN-Nmnat1 expression was confirmed by checking for the coexpressing GFP signal. Cultures were either harvested for RNA extraction or treated with hypoxia (<0.3% O_2_) for 7 h at 37 °C, followed by overnight recovery under normal culture condition. To score for cell death, cultures were stained by ethidium homodimer (Life Technologies; E1169) before fixation. Cells were then stained with Tuj1 (Abcam, Cambridge, UK, AB14545) antibody to identify neurons (green signal shown in Supplementary Figure S1) and with DAPI to identify all cells. At least 10 high-power fields were taken for each infection, and survival values from these fields were pooled as one trial.

### Statistical analysis

GraphPad Prism 6 software (La Jolla, CA, USA) was used for all statistical analysis. Statistical significance was determined by tests indicated in individual figure legends. Paired *T*-tests were used when all trials had concurrent controls; unpaired *T*-tests were used for all other experiments except lifespan comparisons where log-rank tests were performed.

## Figures and Tables

**Table 1:** Summary of strains used in this manuscript

Strain	Parental strain	Injected plasmids	Cell type expression	Method of integration	Coinjection marker
*gcIs29*	N2	P*rpl-28*::*nmat-2*::mCherry	Ubiquitous	UV irradiation	pPHGFP
*gcIs30 IV*	N2	P*rab3*::mCherry::m-nonN-Nmnat1	Pan-neuronal	UV irradiation	pPHGFP
*gcIs31*	N2	P*rab3*::mCherry::m-nonN-Nmnat1	Pan-neuronal	UV irradiation	pPHGFP
*gcIs32*	N2	P*rab3*::mCherry::m-nonN-Nmnat1	Pan-neuronal	UV irradiation	pPHGFP
*gcIs33*	N2	P*rab3*::mCherry::m-nonN-Nmnat1	Pan-neuronal	UV irradiation	pPHGFP
*gcIs34*	N2	P*rab3*::mCherry::m-nonN-Nmnat1	Pan-neuronal	UV irradiation	pPHGFP
*gcIs35*	N2	P*rab3*::mCherry::m-nonN-Nmnat1	Pan-neuronal	UV irradiation	pPHGFP
*gcIs36*	N2	P*rab3*::mCherry::m-nonN-Nmnat1	Pan-neuronal	UV irradiation	pPHGFP
*gcIs37*	N2	P*rpl-28*::*nmat-2*::mCherry	Ubiquitous	UV irradiation	pPHGFP
*gcIs38*	N2	P*rab3*::mCherry::m-nonN-Nmnat1	Pan-neuronal	UV irradiation	pPHGFP
*gcIs39*	N2	P*rab3*::mCherry::m-nonN-Nmnat1	Pan-neuronal	UV irradiation	pPHGFP
*gcIs40*	N2	P*rpl-28*::mCherry::m-nonN-Nmnat1	Ubiquitous	UV irradiation	pPHGFP
*gcIs41*	N2	P*rab3:*:m-nonN-Nmnat1::mCherry	Pan-neuronal	UV irradiation	None
*gcSi1*	EG4322	P*rpl-28*::mCherry::m-nonN-Nmnat1	Ubiquitous	MosSCI	*unc-119(+)*
*gcSi3*	EG6699	P*rpl-28*::nonN-Nmnat1::ERAV2A::nonN-Nmnat1	Ubiquitous	MosSCI	*unc-119(+)*
*gcSi6 II*	EG6699	P*rpl-28*::4x-m-nonN-Nmnat1	Ubiquitous	MosSCI	*unc-119(+)*
*gcSi7 IV*	EG6700	P*rpl-28*::3x-m-nonN-Nmnat1	Ubiquitous	MosSCI	*unc-119(+)*
*gcSi6 II;gcSi7 IV*		7x-m-nonN-Nmnat1	Ubiquitous	MosSCI	*unc-119(+)*
*gcEx145*	N2	P*rpl-28*::*nmat-1*::mCherry	Ubiquitous	Not integrated	pPHGFP

## Abbreviations

*C. elegans*: *Caenorhabditis elegans*
DMSO: dimethyl sulfoxide
EtBr: ethidium bromide
GFP: green fluorescent protein
mitoUPR: mitochondrial unfolded protein response
NAD: nicotinamide adenine dinucleotide
Nmnat: nicotinamide mononucleotide adenylyltransferase
Nmnat1: mouse nicotinamide mononucleotide adenylyltransferase type 1
dNmnat: *Drosophila* nicotinamide mononucleotide adenylyltransferase
m-nonN-Nmnat1: mouse non-nuclear NMNAT1
neuro-m-nonN-Nmnat1: pan-neuronally expressed mouse non-nuclear NMNAT1
ub-m-nonN-Nmnat1: ubiquitously expressed mouse non-nuclear NMNAT1
RNAi: double-stranded RNA-mediated interference
