# Supplementary material for: Nicotinamide mononucleotide adenylyltransferase promotes hypoxic survival by activating the mitochondrial unfolded protein response
Source: Cell Death Dis. 2016 Feb 25;7(2):e2113–. doi: 10.1038/cddis.2016.5 (PMC4849163; doi:10.1038/cddis.2016.5)
Supplement: Supplementary Information [file cddis20165x1.docx]

## Supplementary Information

Figs S1-S6

Tables S1-S2

Supplementary figure legends

**Fig. S1. m-nonN-Nmnat1 expression levels in transgenic strains.** qRT-PCR(SYBR green, ΔΔC_T_ method) was used to assess the m-nonN-Nmnat1 transcript levels in the various strains. The relative mRNA values were referenced to g*cSi1* which was set as 1. Values are mean ± SEM.

**Fig. S2. m-nonN-Nmnat1 or NMAT-2 expression did not affect O_2_ consumption.** Neuro-Nmnat1(*gcIs30*[Neuro-m-nonN-Nmnat1], Ub-nmat-2(*gcIs37*[Ub-nmat-2]), Ub-m-nonNmnat1(*gcIs40*[Ub-m-nonN-Nmnat1] -Values are mean±SEM, p>0.05 between any two groups, unpaired T-test, n=3-9 trials.

**Fig. S3. Screening for pathways required for m-nonN-Nmnat1 hypoxic protection**. (**a)** *skn-1, hsf-1, trx-1* mutants did not suppress Neuro-Nmnat1(*gcIs30*[Neuro-m-nonN-Nmnat1]) increased hypoxic survival. (**b**) Mutation of the ER-UPR genes *ire-1* and *xbp-1* did not suppress Neuro-Nmnat1(*gcIs30*[Neuro-m-nonN-Nmnat1]) hypoxic survival. (**c**) Multiple MAP kinase pathways were not required for Neuro-Nmnat1(*gcIs30*[Neuro-m-nonN-Nmnat1]) hypoxic survival. (All values are mean±SEM, minimal trial number 3; none of the mutations significantly reduced hypoxic survival, paired T-test, p>0.05)

**Fig. S4. Other ABC transporters and mitoUPR components were not required for m-nonN-Nmnat1 hypoxic protection**. (**a**) Reduction of the function of mitochondrial proteases did not suppress Neuro-Nmnat1(*gcIs30*[Neuro-m-nonN-Nmnat1]) hypoxic survival (n=3). (**b**)Mutation of the ABC transporter homologs *haf-2* and *haf-3* did not suppress Neuro-Nmnat1(*gcIs30*[Neuro-m-nonN-Nmnat1]) hypoxic survival (n=5, paired T-test).

**Fig. S5. nonN-Nmnat1 induces mitochondrial chaperone expression in mouse primary hippocampal neurons.** Mouse hippocampal cultures were transduced by lentivirus coding mCherry (control), nonN-Nmnat1-mCherry (also known as cytNmnat1), or nonN-Nmnat1-mCherry (H24A) (Enzymatic activity is compromised in H24A mutation). Five days later, RNA was extracted and the transcript levels of hspa9 (closest *hsp-6* homolog, open bars) and hspd1 (closest *hsp-60* homolog, black bars) were determined by qRT-PCR. (n= 3 trials, **p<0.01 vs control or H24A, unpaired T-test)

**Fig. S6. Taxol and low copy Neuro-Nmnat1 (*gcIs35*[Neuro-m-nonN-Nmnat1]did not alter worm life span.** Taxol [3μM] was present in the whole course of the life span analysis. FUDR [100μM] was added in the first week of adult stage to suppress matricide.
