# Supplementary figures and images for: Nicotinamide mononucleotide adenylyltransferase promotes hypoxic survival by activating the mitochondrial unfolded protein response
Source: Cell Death Dis. 2016 Feb 25;7(2):e2113–. doi: 10.1038/cddis.2016.5 (PMC4849163; doi:10.1038/cddis.2016.5)

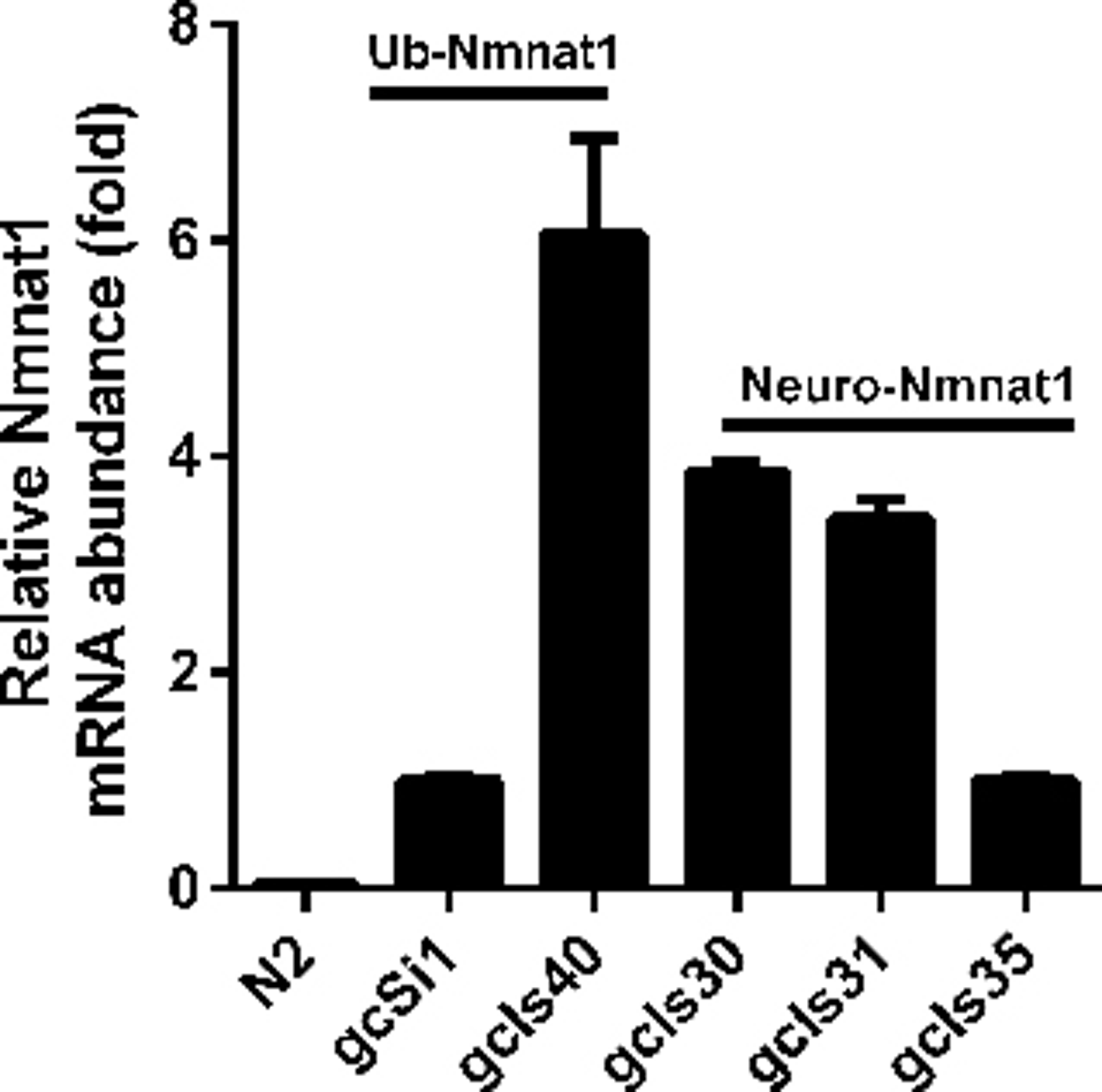

Supplement: Supplementary Figure 1 [file cddis20165x2.tif]

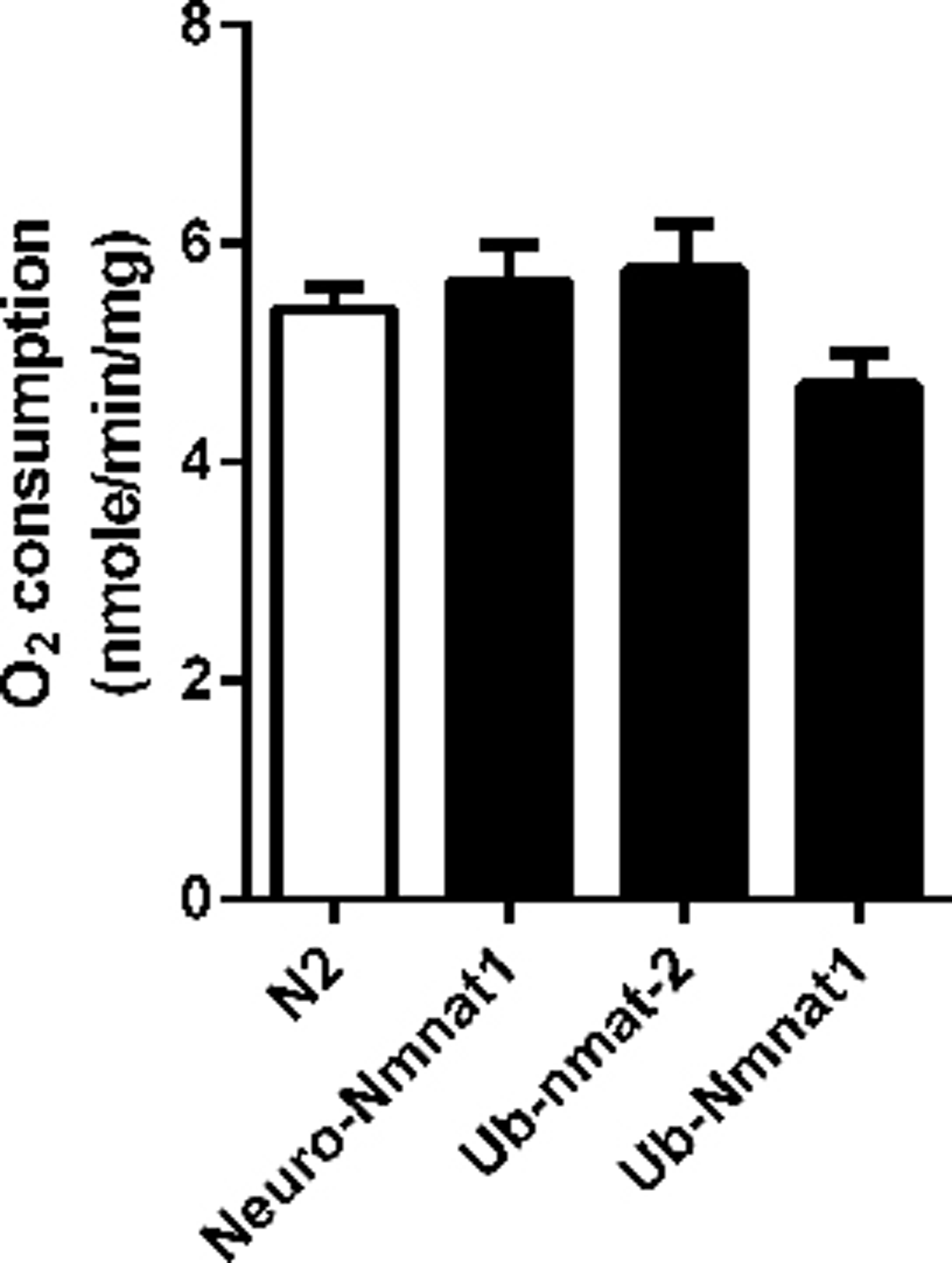

Supplement: Supplementary Figure 2 [file cddis20165x3.tif]

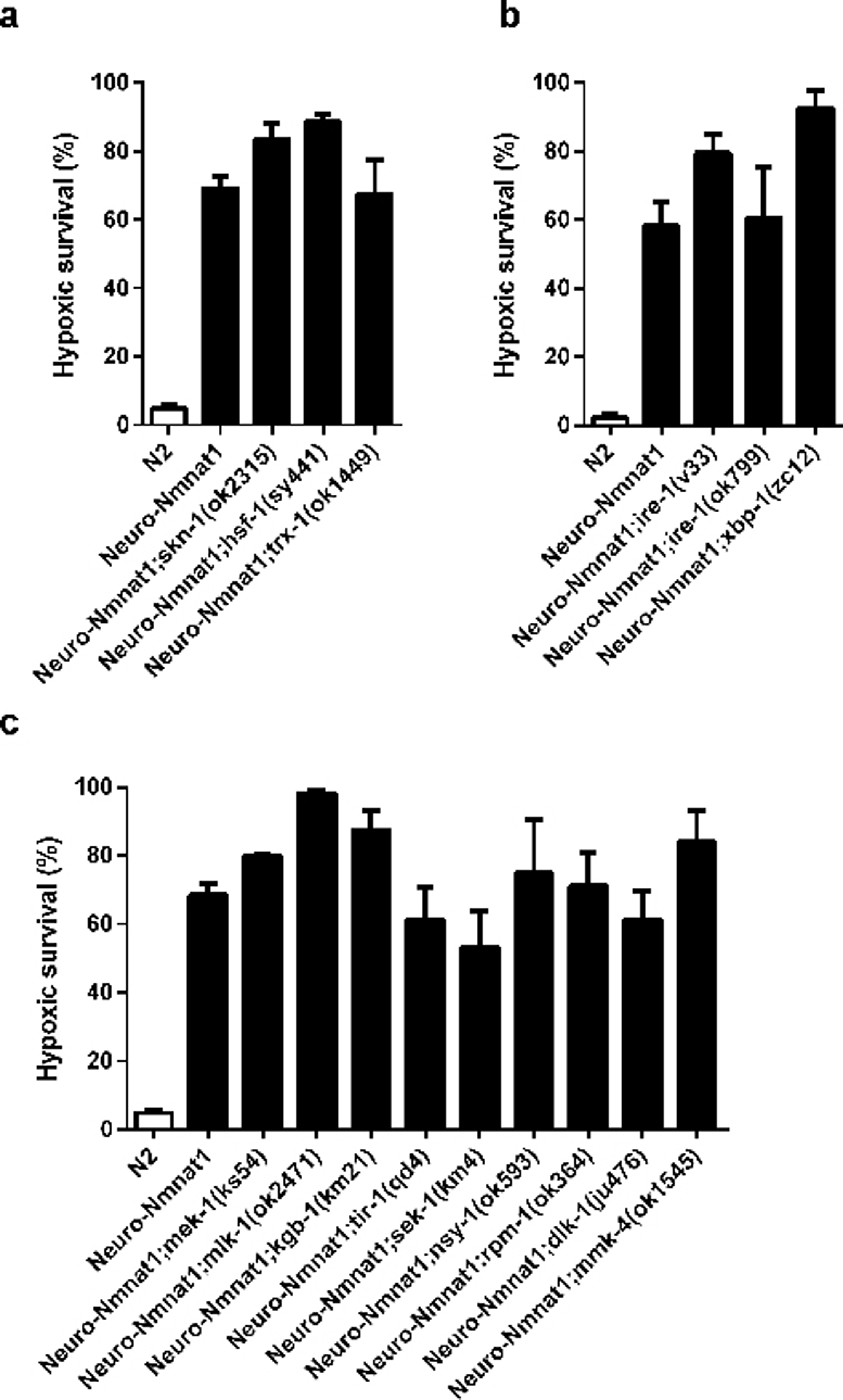

Supplement: Supplementary Figure 3 [file cddis20165x4.tif]

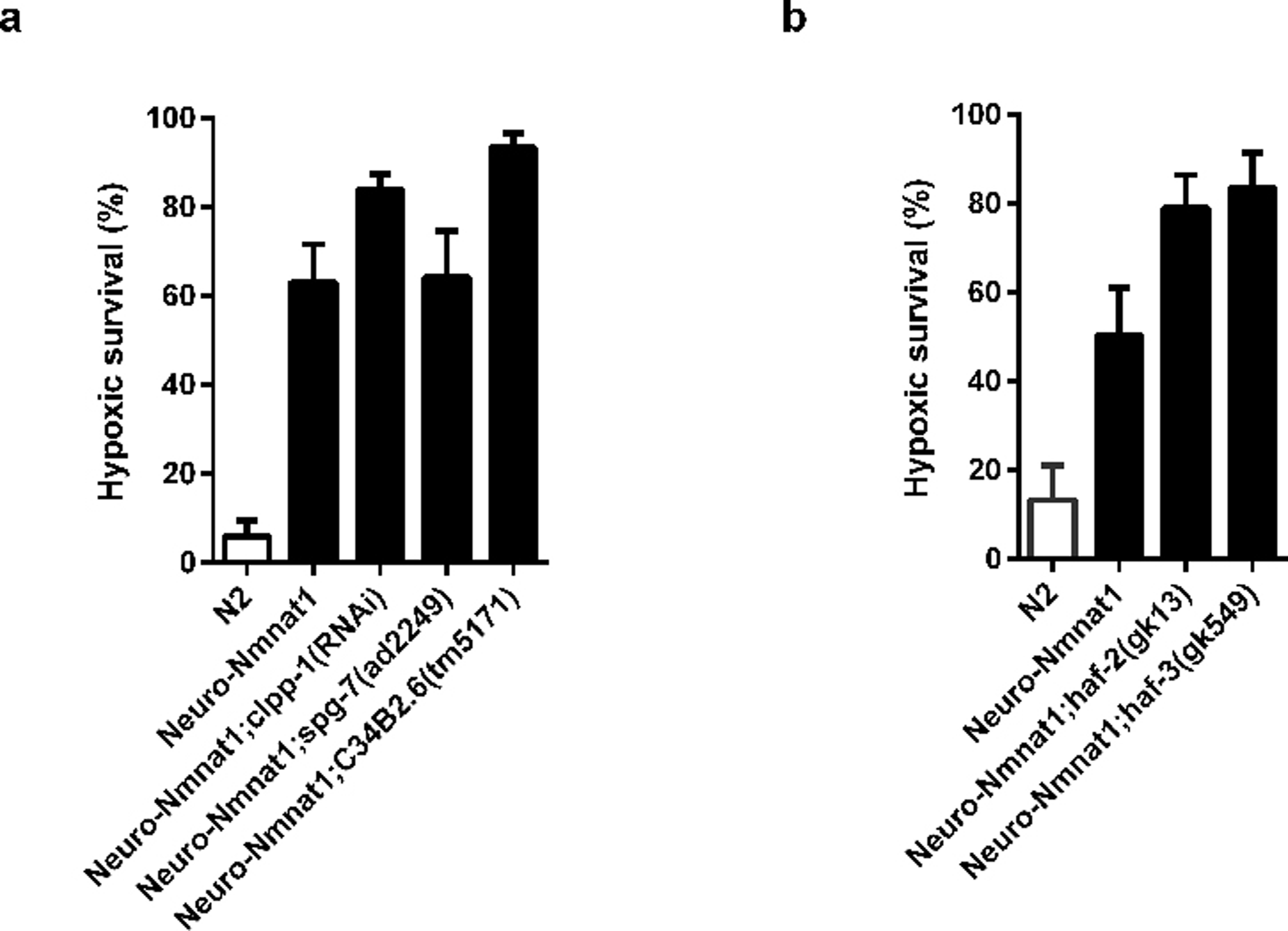

Supplement: Supplementary Figure 4 [file cddis20165x5.tif]

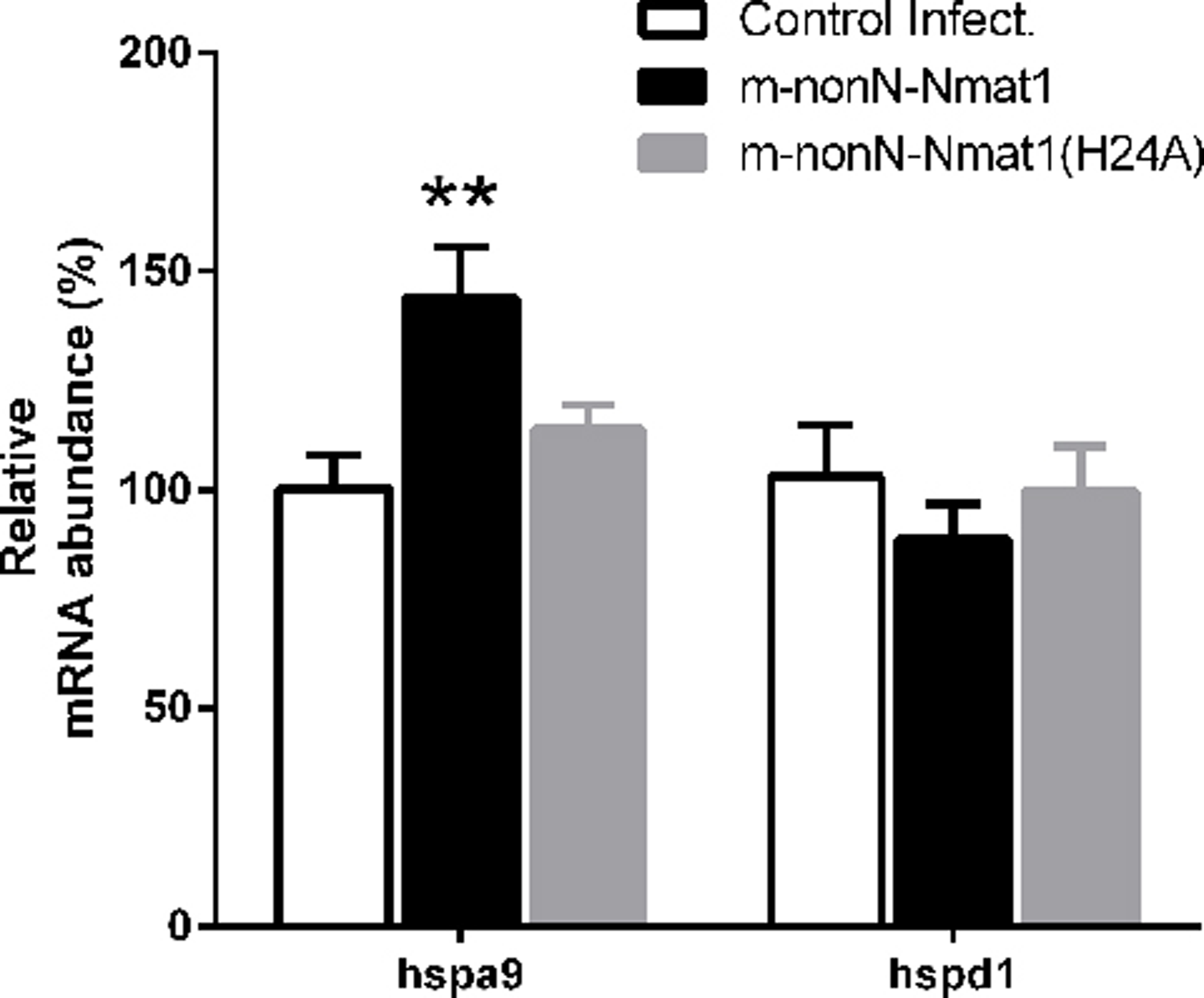

Supplement: Supplementary Figure 5 [file cddis20165x6.tif]

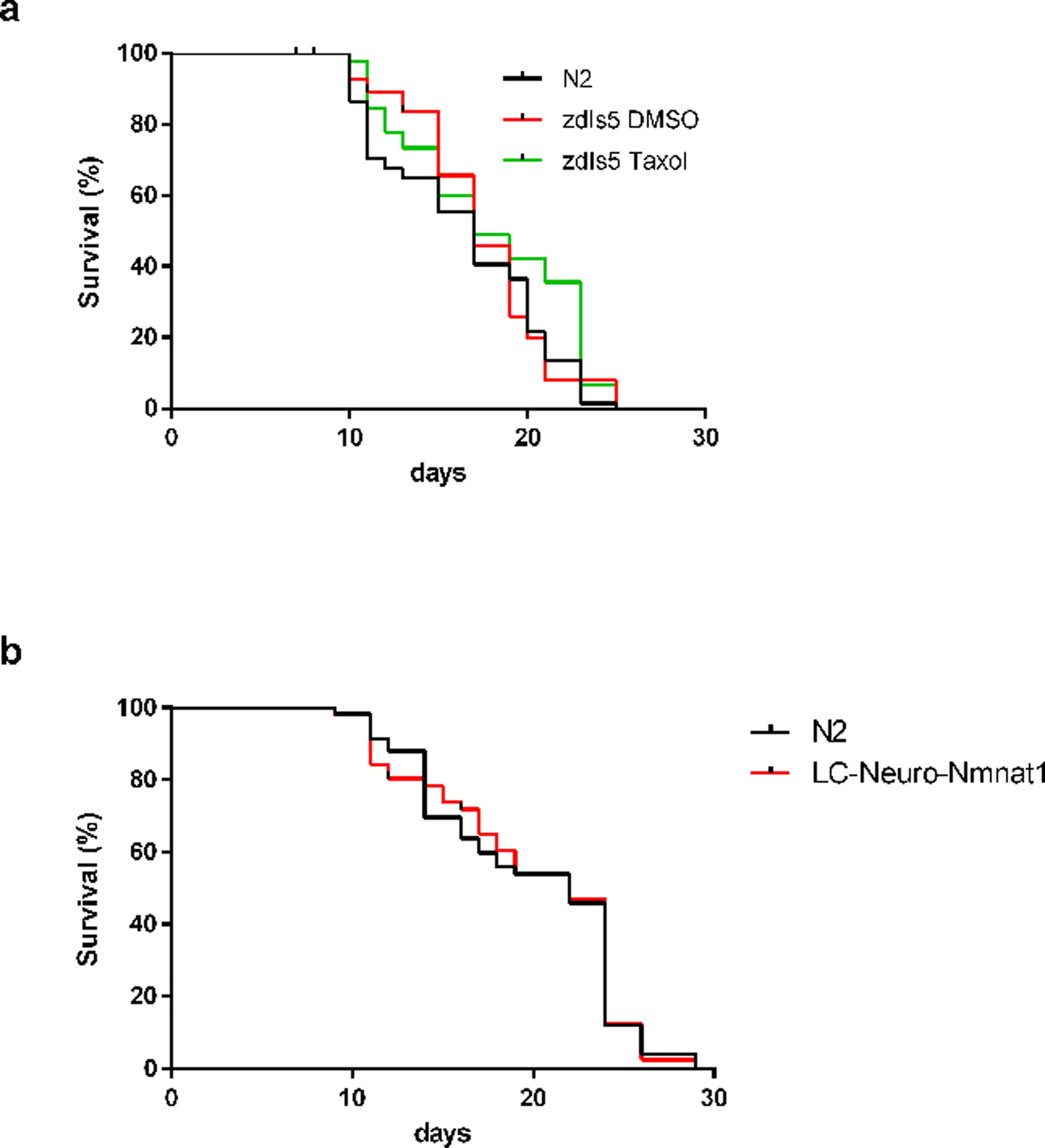

Supplement: Supplementary Figure 6 [file cddis20165x7.tif]
